# Do parents’ support behaviours predict whether or not their children get sufficient sleep? A cross-sectional study

**DOI:** 10.1186/s12889-017-4334-4

**Published:** 2017-05-24

**Authors:** Evelyn Pyper, Daniel Harrington, Heather Manson

**Affiliations:** 1 Public Health Ontario, 480 University Avenue, Suite 300, Toronto, ON M5G 1V2 Canada; 2 School of Public Health and Health Systems, University of Waterloo, 200 University Avenue West, Waterloo, Toronto, ON N2L 3G1 Canada; 3 Dalla Lana School of Public Health, University of Toronto, 155 College Street, 6th floor, Toronto, ON M5T 3M7 Canada

**Keywords:** Parent support behaviours, Sleep, Screen time, Healthy child development, Overweight and obesity

## Abstract

**Background:**

Sleep is an essential component of healthy cognitive and physical development. Lack of sleep may put children at risk for a variety of mental and physical health outcomes, including overweight, obesity and related chronic diseases. Given that children’s sleep duration has decreased in recent decades, there is a need to understand the determinants of child sleep, including the role of parental support behaviours. This study aims to determine the relative contribution of different types of parental support behaviours for predicting the likelihood that children meet recently established Canadian sleep guidelines.

**Methods:**

Data were collected using Computer Assisted Telephone Interviews (CATI) of parents or guardians with at least one child under the age of 18 living in Ontario, Canada. To align with sleep guidelines, parents included in this analysis had at least one child between 5 and 17 years of age (*n* = 1622). Two multivariable logistic regression models were built to predict whether or not parents reported their child was meeting sleep guidelines – one for weekday sleep and another for sleep on weekends. Independent variables included parent and child age and gender, motivational and regulatory parental support behaviours, and socio-demographic characteristics.

**Results:**

On weekdays, enforcing rules about child bedtime was a significant positive predictor of children meeting sleep guidelines (OR: 1.59; 95% CI: 1.03–2.44); while encouraging the child to go to bed at a specific time was a significant negative predictor of child meeting sleep guidelines (OR: 0.29; 95% CI: 0.13–0.65). On weekends, none of the parental support behaviours contributed significantly to the predictions of child sleep. For both weekdays and weekends, the child’s age group was an important predictor of children meeting sleep guidelines.

**Conclusions:**

The contribution of parental support behaviours to predictions of children meeting sleep guidelines varied with the type of support provided, and weekend versus weekday sleep. While only enforcing bedtime rules on weekdays contributed to children meeting sleep guidelines, the importance of children getting a good night’s sleep, and the capacity of parents to help them do so, should be emphasized in public health efforts promoting healthy child development.

## Background

Lack of sleep has become increasingly recognized as a contributor to the obesity epidemic in children and adolescents [1]. Elevated rates of childhood obesity and overweight in western countries within the last decade [2–4] have garnered substantial attention from public health, with a particular emphasis on promoting healthy active living, and limiting sedentary behaviour. While both sedentary behaviour and sleep involve low energy expenditure, they have opposite effects on children’s weight [5]. Sleep is an essential component of healthy cognitive and physical development, and lack thereof can negatively impact children’s physical activity levels as well as hormones associated with increased risks of obesity, diabetes and hypertension [1, 6–8]. Even after controlling for other known risk factors, inadequate sleep significantly increases the risk of pediatric obesity [9, 10]. Shorter sleep duration has also been shown to be associated with poorer emotional regulation, academic achievement, and lower quality of life/well-being [11].

Child sleep and family functioning (e.g., marital conflict; parenting stress) are inextricably linked [12–14], and thus, it is imperative to view sleep from a family context [15–17]. Indeed, behavioural interventions in toddlers and preschoolers have proven effective for improving both child sleep and family functioning [18, 19]. It follows that parents’ behaviours play a key role in influencing children’s sleep patterns. Although the types of behaviours that support adequate sleep may be more limited than those for other health behaviours, type of support has been shown to be important for achieving child sleep outcomes. For example, periodically checking on the child has been associated with longer sleep duration in 11-year-olds [20]; conversely, active physical comforting of one- and two-year-old children has been associated with children’s sleep problems (e.g., resisting bedtime, waking at night) [15]. This highlights the need to clearly discern which parental support behaviours for which age groups are optimal for child sleep.

Previous research has found that parents can actively encourage their child’s development of self-regulation [21], and this encouragement of autonomy and independence has been linked to fewer sleep problems and more consolidated child sleep [22, 23]. Parents can also support their child’s sleep through enforcing rules, such as setting bedtimes and structuring the child’s evening time [14]. Although sleep needs and challenges differ greatly across children’s ages, establishment of a bedtime routine and development of a structured environment (e.g., regular family mealtimes) may improve sleep for both young children and adolescents [14, 24, 25]. Moreover, sleep quality and duration may be negatively impacted by media use, with a particular threat being screen time in the bedroom [7, 26, 27]. Parents’ restriction or discouragement of nighttime media use has the potential to foster healthy child sleep behaviours [26, 28], although determining the best means of doing so remains elusive.

Children’s sleep duration has decreased by about 30 to 60 min in recent decades [29, 30], and in the Canadian context, recent estimates reveal that 31% of school-aged kids and 26% of adolescents are sleep-deprived [31]. In acknowledgement of the emerging “sleepidemic,” the 2016 ParticipACTION Report Card on Physical Activity for Children and Youth brought sleep to the forefront of discussion of inactivity in Canadian children [32]. This most recent report also promotes the new *Canadian 24-Hour Movement Guidelines for Children and Youth* [33], which outlines what a healthy 24-h period should look like for 5- to 17-year-olds. The guidelines introduce a new movement paradigm—of which sleep is an integral component—emphasizing a whole day approach. It presents sleep, not only as an important behaviour in its own right, but also as highly integrated with the other movement behaviours of physical activity and sedentary behaviour. While there is mounting evidence of the importance of sleep as a protective factor for childhood overweight and obesity, there remains uncertainty surrounding the optimal role of parental support for child sleep [15].

### Objectives

The objective of this study is to determine the relative contribution of different types of parental support behaviours for predicting the likelihood that children meet sleep guidelines on weekdays and weekends.

## Methods

### Data

This study uses data collected from a Computer Assisted Telephone Interview (CATI) of Ontario parents/guardians with at least one child under the age of 18 (*n* = 3206). Details of the survey methodology can be found elsewhere [34]. Briefly, the CATI data were collected by Public Health Ontario between February and March, 2015. The survey was developed for the evaluation of a community-based program targeting childhood overweight and obesity. To randomize at the household level, parents were asked to respond on behalf of the child in the household with the next birthday. This study is a secondary analysis of a subsample baseline data, and it specifically focuses on parents who answered questions in the survey about sleep. Moreover, parents included in the subsample had at least one child between 5 and 17 years of age, in order to make comparisons with established sleep guidelines (*n* = 1622).

The dependent variables in this study were derived from survey questions asking parents: (i) what time their child usually wakes up during the week; (ii) what time their child usually gets to bed during the week; (iii) what time their child usually wakes up on the weekend; and (iv) what time their child usually gets to bed on the weekend. Bedtimes and wake-up times were used to calculate total minutes of sleep duration for weekdays and weekends. Sleep duration was compared to the *Canadian 24-Hour Movement Guidelines for Children and Youth* [33] (Table 1), accounting for child age, in order to construct binary dependent variables (i.e., meets guideline versus does not meet guideline) for weekdays and weekends. Children who were below and above the sleep duration recommendations were classified as not meeting the guideline.

**Table 1:** Sleep guidelines from the *Canadian 24-Hour Movement Guidelines for Children and Youth*

Age	5–13 years	14–17 years
Guideline	Uninterrupted 9 to 11 h of sleep per night	Uninterrupted 8 to 10 h of sleep per night

The four parental support behaviours for child sleep represent key independent variables of this study. Parents were asked to indicate whether they *strongly agree*, *agree*, *neither agree or disagree*, *disagree*, or *strongly disagree* with each of the statements found in Table 2. The selection of bedtime- and screen-related support behaviours reflects evidence that: family rules are related to earlier bedtimes and greater total hours of sleep [14]; screen time of children (and parents’ control over that screen time) also influence child sleep duration [26–28]. Dividing the support behaviours into “encouragement” and “rule enforcement” reflects the need to clearly discern which specific types of parental support predict children meeting sleep guidelines, as there is a paucity of research making this distinction.

**Table 2:** Parental support behaviours for child sleep, by type of support

	Motivational	Regulatory
Bedtime	I encourage my child to go to bed at specific time	I enforce rules about my child’s bedtime
Screens	I encourage my child to limit the time spent looking at screens in their bedroom	I enforce rules about having screens in my child’s bedroom

Accordingly, the second level of categorization as *Motivational* or *Regulatory* (Table 2), is based on an adapted framework [34] for classifying parental support behaviours proposed by Beets et al. [35] that aimed to answer analogous questions about parental support in the context of physical activity, healthy eating, and screen time. To create binary independent variables, the responses *strongly agree* and *agree* were collapsed into a single item, as were the remaining responses.

While derivations of the other independent variables are described elsewhere [34], they are also provided here. For the logistic regression models, child age was re-coded from a continuous variable to a categorical variable with the following age groups: 5 to 9 years old, 10 to 13 years old, and 14 to 17 years old. A binary variable was created for parental marital status, comprised of *living with partner* (living common-law, married) and *not living with partner* (widowed, separated, divorced, single). Time since immigration was re-coded to become *0 to 9 years* (0–4 years ago, 5–9 years ago), *10+ years* (10–20 years ago, more than 20 years ago), and *Canadian-born*. A variable measuring parents’ highest level of education completed was re-coded as follows: *below secondary school* (never attended school, less than secondary school graduation), *secondary school diploma*, and *post-secondary school* (apprenticeship or trades certificate or diploma; college, CEGEP or other non-university certificate or diploma; university certificate or diploma below bachelor level; bachelor’s degree; university certificate, diploma or degree above bachelor level). Employment status was also re-coded into the following categories: *employed* (full-time, part-time, self-employed), *unemployed*, and *other* (Ontario Disability Support Program, retired, student, homemaker, working without pay, other). Total household income categories were re-coded from increments of $10,000 to increments of $20,000. It should be noted that those who refused to report their income were treated as their own category (*did not respond*).

### Statistical analysis

All analyses were conducted with SAS Version 9.3. Descriptive statistics were calculated to describe the sample by demographic characteristics, parental support behaviours, and child sleep duration. Bivariate analyses were used to preliminarily consider the relationships between all independent variables and dependent variables (i.e., whether children meet the guidelines) to assess unadjusted associations, and inform how the multivariable logistic regression models were to be built.. Specifically, independent samples t-tests were conducted for continuous variables, and chi-square tests were conducted for categorical variables. Independent variables included: child age and gender, parent age and gender, marital status, time since immigration, education, employment status, and total household income. Variance inflation factors indicated that collinearity was not a concern.

To determine the relative importance of parent support behaviours in predicting whether children meet the *Canadian 24-Hour Movement Guidelines for Children and Youth* for sleep [33], two multivariable logistic regressions were used—one for weekdays and one for weekends. Models were built starting with parent and child age and gender; followed by the inclusion of parental support behaviours; followed by demographic characteristics. Interaction terms for all combinations of child age and parental support behaviours were tested. While some significant interactions emerged, interpretation of the estimates was not intuitive. As such, interaction terms were ultimately excluded to present more parsimonious models, and minimize the risk of interpreting spurious results. Differences between estimates were tested for statistical significance at *p* < 0.05.

## Results

### Sample

Children ranging from 5 to 17 years old (mean = 11.1, sd = 3.8, median = 11) were proportionately comprised of males (*n* = 830, 51.2%) and females (*n* = 792, 48.8%). The majority of parents responding to the survey were female (72.4%), and parents’ ages ranged from 24 to 73 years (mean = 44.4, sd = 7.3, median = 44). Demographic characteristics and other variables describing the study sample can be found in Table 3. It should be noted that counts for missing categories—including those who responded “Don’t know” and those who refused to respond—are not included in all tables.

**Table 3:** Characteristics of the study sample

	Sample		Ontario Population^a^	
	*n* = 1622			
Variable	N	%	N	%
Child gender				
Male	830	51.2	1,381,630	51.3
Female	792	48.8	1,312,220	48.7
Child age category (years)^b^				
5 to 9	619	38.2	712,755	35.8
10 to 17	1003	61.8	1,276,840	64.2
Parent gender				
Male	447	27.6	--	--
Female	1174	72.4	--	--
Parent age (Mean ± SD)	44.4 ± 7.3		--	
Marital status				
Not living with partner	260	16.2	604,645	16.7
Living with partner	1349	83.8	3,007,560	83.3
Time since immigration				
Canadian-born	1247	78.0	8,906,000	71.1
10+ years	277	17.3	2,591,915	20.7
0–9 years	75	4.7	1,019,460	8.1
Education, highest level completed				
Below secondary school	46	2.9	769,575	11.0
Secondary school diploma	187	11.6	1,702,160	24.3
Post-secondary school	1379	85.6	4,547,145	64.8
Employment status				
Employed	1309	81.4	16,595,030	60.9
Unemployed	70	4.4	1,395,050	5.1
Other	230	14.3	9,269,445	34.0
Total household income^c^				
Respondents	1309	100	4,886,655	100
< $20,000	51	3.1	556,305	11.4
$20,000 -$39,000	103	6.4	831,135	17.0
$40,000 -$59,000	162	10.0	824,425	16.9
$60,000–$79,000	167	10.3	680,850	13.9
$80,000–$99,000	213	13.1	552,660	11.3
≥ $100,000	613	37.8	1,441,280	29.5

^a^All Ontario data retrieved from: Statistics Canada, 2011 Census of Population

^b^Child age categories presented are based on available census data

^c^Total household income, before taxes and deduction, from all sources in the past 12 months. Note that 21.1% of the study sample did not provide their total household income

-- Comparable data for the Ontario population not available (e.g., Census does not differentiate between adults and parents; thus, parent-related data cannot be retrieved).

#### Guidelines

**Fig. 1:**
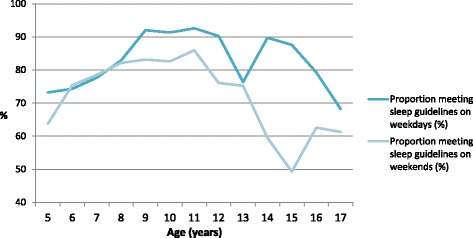
Proportion of children meeting the *Canadian 24-Hour Movement Guidelines for Children and Youth* for sleep on weekdays and weekends by child age, as reported by parents

The proportion of parents that reported their child was meeting the sleep guidelines ranged from 68.3% to 92.6% for weekdays, and 49.3% to 86.0% for weekends. As shown in Fig. 1, the average proportion of children of each age meeting sleep guidelines appeared to increase between 5 and 9 years of age, and decline thereafter. In addition to the decline in meeting sleep guidelines seen between 9 and 17 years of age, this age range also appears to be where discrepancy exists between weekdays and weekends. The greatest difference is seen for 15 year-olds, where the average proportion of children meeting sleep guidelines on weekends is 38.3% less than on weekdays.

#### Parental support Behaviours

The proportion of parents engaging in support behaviours for sleep were as follows: 93.9% encourage their child to go to bed at a specific time; 89.6% encourage their child to limit the time spent looking at screens in their bedroom; 84.4% enforce rules about their child’s bedtime; and 77.4% enforce rules about having screen’s their child’s bedroom.

**Fig. 2:**
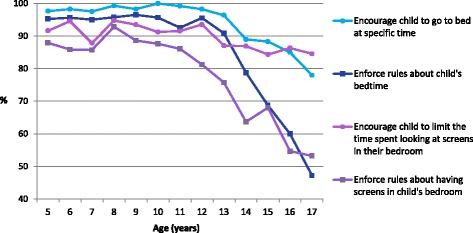
Proportion of parents reporting engaging in different support behaviours for sleep, by child age

As Fig. 2 depicts, the proportion of parents engaging in support behaviours for child sleep decreased between the ages of 12 and 17, approximately. The largest decline in these adolescent years was seen for the *regulatory* support behaviours (enforcing rules).

#### Weekday model

As shown in Table 4, the following parental support behaviours contributed significantly to the predictions of child sleep on weekdays: parents who reported encouraging their child to go to bed at a specific time were 71% *less* likely to have their child meeting sleep guidelines (OR: 0.29; 95% CI: 0.13–0.65); while parents who reported enforcing rules about their child’s bedtime were 1.59 times *more* likely to have their child meeting sleep guidelines (95% CI: 1.03–2.44). These associations remained significant in the model after adjusting for demographic and socioeconomic characteristics. Moreover, compared to 10- to 13-year-old children, 5- to 9-year-old children were 45% less likely to meet sleep guidelines on weekdays (95% CI: 0.37–0.80), while 14- to 17-year-olds were 33% less likely (95% CI: 0.44–1.00). Looking at the association of predicted probabilities and observed responses, the c-statistic, or area under the receiver operating characteristic (ROC) curve is 0.63, indicating a reasonably good model fit.

**Table 4:** Multivariable logistic regression predicting the likelihood that parents report their child meets sleep guidelines on weekdays, adjusted for demographic and socioeconomic characteristics

Effect	Odds ratio point estimate	95% Wald confidence limits		*p-value*
Intercept	--	--	--	0.0140*
Child age categories (years)				
5 to 9	0.55	0.37	0.80	0.0017**
10 to 13	1.00			
14 to 17	0.67	0.44	1.00	0.0498*
Child gender				
Male	1.01	0.76	1.33	0.9512
Female	1.00			
Parent age	1.00	0.98	1.03	0.9095
Parent gender				
Male	0.98	0.71	1.36	0.9186
Female	1.00			
Encourage child to go to bed at specific time	0.29	0.13	0.65	0.0025**
Enforce rules about child’s bedtime	1.59	1.03	2.44	0.0354*
Encourage child to limit the time spent looking at screens in their bedroom	1.33	0.84	2.10	0.2302
Enforce rules about having screens in child’s bedroom	1.12	0.77	1.64	0.5430
Association of Predicted Probabilities and Observed Responses				
Area under the ROC curve (c) = 0.63				

Significance code: *p < 0.05, **p < 0.01

#### Weekend model

As shown in Table 5, none of the parental support behaviours contributed significantly to the predictions of child sleep on weekends. 14- to 17-year olds were 74% less likely to meet sleep guidelines on weekends than those aged 10- to 13-year-old (95% CI: 0.19–0.37). Male children were 1.28 times more likely to meet sleep guidelines on weekends than female children (95% CI: 1.01–1.62). Moreover, for parent age, every additional year increased the likelihood of a child meeting sleep guidelines on weekends by 3% (95% CI: 1.01–1.05). Looking at the association of predicted probabilities and observed responses, the c-statistic, or area under the ROC curve is 0.68, indicating a reasonably good model fit.

**Table 5:** Multivariable logistic regression predicting the likelihood that parents report their child meets sleep guidelines on weekends, adjusted for demographic and socioeconomic characteristics

Effect	Odds ratio point estimate	95% Wald confidence limits		*p-value*
Intercept	--	--	--	
Child age categories (years)				
5 to 9	0.79	0.57	1.11	0.1741
10 to 13	1.00			
14 to 17	0.26	0.19	0.37	<.0001***
Child gender				
Male	1.28	1.01	1.62	0.0456*
Female	1.00			
Parent age	1.03	1.01	1.05	0.0062**
Parent gender				
Male	0.85	0.64	1.12	0.2367
Female	1.00			
Encourage child to go to bed at specific time	1.22	0.73	2.05	0.4428
Enforce rules about child’s bedtime	0.78	0.54	1.13	0.1934
Encourage child to limit the time spent looking at screens in their bedroom	0.93	0.62	1.41	0.7387
Enforce rules about having screens in child’s bedroom	1.11	0.81	1.53	0.5232
Association of Predicted Probabilities and Observed Responses				
Area under the ROC curve (c) = 0.68				

Significance code: *p < 0.05, **p < 0.01, ***p < 0.001

## Discussion

A major finding of the current study is that only parental support in the form of weekday bedtime rule enforcement contributed significantly to children meeting sleep guidelines; however, findings surrounding child age and weekday/weekend differences also warrant further discussion. Our results suggest that the proportion of children meeting Canadian sleep guidelines decreases as they enter and progress through adolescence. This discovery is consistent with contemporary sleep duration estimates by Chaput and Janssen [31], which reveal shorter sleep durations in Canadian children and adolescents as they grow older. The sleep duration trend may be an indication of later bed times, rather than earlier wake up times. For instance, almost half of 16- to 17-year-olds reported going to bed after midnight on weekends [31]). A number of factors contributing to insufficient sleep in adolescents have been identified, including: (1) biological processes (i.e., an evening-type circadian phase; delayed melatonin production), (2) social obligations (i.e., early school start times), (3) consumption of caffeine (i.e., soda, coffee, and energy drinks), and (4) media use (i.e., direct displacement of sleep; disruption of circadian rhythms by light; or increased sleep-disrupting arousal) [36].

The present study compared the proportion of children meeting sleep guidelines on weekdays and weekends, and found the greatest difference in 15-year-olds. Interestingly, results from a 2014 multicohort study of over 270,000 American adolescents observed that over the previous 20 years, the largest decrease in the proportion getting ≥7 h of sleep was also in 15-year-olds [29]. As a potential high-risk group, the biological and social factors at play for this particular age may warrant further elucidation.

The larger discrepancies between weekday and weekend sleep habits seen from middle childhood through adolescence echo the work of others [7]. It is well established that adolescents accumulate sleep debt from regularly going to bed later during the week, which may lead to later wake times on weekends (also known as catch-up sleep) [29–31, 36, 37]. Yet, what appears inconsistent is whether this sleep debt leads to longer [11] or shorter (as found in the present study) weekend sleep duration in adolescents.

The proportion of parents engaging in support behaviours for sleep was lower for the two *regulatory* support behaviours, relative to the two *motivational* support behaviours. Similarly, the largest decline in parental support for adolescents of increasing age was seen for the two *regulatory* support behaviours. As children get older, parents are usually less involved with their sleep routines [17]. Nevertheless, parental rules—including an earlier bedtime—have been shown to increase adolescent total sleep [14], highlighting the need for increased *regulatory* parental support.

In the weekday model, two parental support behaviours contributed significantly to predictions of children meeting sleep guidelines. First, parents who reported encouraging their child to go to bed at a specific time were less likely to have children meeting sleep guidelines on weekdays. Given that *motivational* support behaviours are defined by “prompting others to engage in the behaviour of interest” [35], it was unexpected to find that encouragement was a negative predictor of sufficient sleep in children. Because of this study’s cross-sectional design, we cannot discern the direction of the relationship between parental support and child sleep. Accordingly, one possible explanation for this finding is that “encouraging one’s child to go to bed at a specific time” is a parent’s reaction to their child’s already-established poor sleep habits. In other words, encouragement may lend itself to being a reactive behaviour, while other behaviours—such as rule-setting—may be more proactive. Given the lack of research on parental support for child and adolescent sleep, the proposed explanations would require further investigation in order to be substantiated.

Second, parents who reported enforcing rules about their child’s bedtime were more likely to have their child meeting sleep guidelines on weekdays. Family rules have been shown to be associated with earlier bedtimes and greater total hours of sleep on weekdays for older children (12 to 19 years old) [14]. One reason for this association proposed by Adam, Snell, & Pendry [14] is that parents more strictly enforce rules regarding bedtimes during the week; however, their results also suggest that it is not merely stricter bedtime rules—but rather more general parental expectations, structure, or monitoring—that affect child sleep on weekdays. In a meta-analysis of risk and protective factors associated with adolescent sleep, parent-set bedtime had the greatest positive correlation with sleep duration [38]. However, there is evident dissonance between parental bedtime rule-setting as an important determinant of adolescent sleep, and as a parent support behaviour reported less frequently as children enter adolescence. The present study includes a range of child ages—not one particular age—when looking at the effect of bedtime rule enforcement on child sleep. Age-specific investigations are warranted to determine if these findings hold true in different age groups. Future studies should also consider which specific rules parents can set and how parents can enforce these rules to best support child sleep.

In the weekend model, none of the parental support behaviours contributed significantly to predictions of children meeting sleep guidelines. While non-significant results for *all* support behaviours was unexpected, this finding parallels that of prior research showing family variables as better predictors of weekday, as oppose to weekend, sleep behaviours [14]. A potential explanation for these results is that parents exercise less control over children’s schedules on weekends, giving children more discretionary time to choose what activities they engage in [39]. Time spent watching television, using the computer or video games, socializing, playing sports, working, and participating in religious activities all predict fewer hours of sleep on weekends [14]. Our results do not rule out parental support behaviours as important determinants of child sleep on weekends; instead, they point to the need to explore the child’s social and household environments to gain a full picture of the many factors affecting weekend sleep behaviours.

A noteworthy finding of the current study is that, while the use (and even presence) of screens in the bedroom can negatively affect children’s sleep [40, 41], parental rule enforcement and encouragement to limit screens in the bedroom did not predict children meeting sleep guidelines on weekdays or weekends. One possible explanation is that use of electronic media or “screen time” is an unstructured leisure activity, with no clear starting and ending point [27]. Use of screens in the bedroom may represent a child behaviour over which parents have limited control [26]. Another contributing factor is that restricting media may make it all the more enticing [42], and the child’s bedroom may be a location where screen use is less supervised. Finally, it is important to acknowledge the role of not only a parent, but the entire family, in the creation and perpetuation of a child’s sleep-displacing screen use [26]. Our findings draw attention to the notion that traditional parenting behaviours (rule-setting, encouragement) may not be sufficient when it comes to changing screen time behaviours in the bedroom; the sleep and screen use behaviours and expectations of the entire family should considered in future studies.

### Limitations

The present study has several limitations that warrant discussion. First, all information was obtained from survey data, and therefore, measures of parental support and child sleep were parent-reported. Both measures are therefore subject to recall bias, as well as social desirability bias stemming from the desire to appear as a supportive parent. In particular, child sleep duration may be overestimated if parents’ responses were based on when their child was *in bed*, as opposed to when their child was actually *asleep*. Despite the drawbacks of using subjective versus objective measures, current sleep duration recommendations are based largely on self-reported sleep data [43]. A second, related limitation is the study’s use of sleep duration as measure of children’s sleep health. While duration is an important component of sleep hygiene, other aspects including sleep quality, consistency, continuity, and timing play an important part in one’s overall health and well-being [32]. The *Canadian 24-Hour Movement Guidelines for Children and Youth* specify “uninterrupted” sleep; however, because sleep continuity could not be measured, the use of sleep duration data allowed for clear comparisons with these sleep recommendations in order to construct the primary outcome variable. It should still be noted that because this study’s measure of child sleep is based on parent report, we cannot know whether the hours of sleep are fragmented or truly uninterrupted. Third, parents were asked the extent to which they agreed with statements starting with “I encourage…” and “I enforce rules…” (Table 2) without being provided with definitions of encouragement and rule enforcement. Thus, parents’ perceptions of these words informed the measures of parental support for child sleep. Fourth, while the logistic regression models of the present study controlled for factors such as time since immigration, education, employment status, and total household income, other cultural factors were not included. Although it was beyond the scope of this study to ascertain cultural backgrounds and implications for sleep habits, it is important to acknowledge that cross-cultural variation exists for sleep timing and duration [44]. Fifth, variables exploring the whole day of the child (e.g., physical activity and sedentary time) were not able to be included in the analyses. Due to the length of the survey, parents were asked to answer at least two of the four health behaviour modules (physical activity, healthy eating, recreational screen time, and sleep). These were randomly selected at the time of the interview. Participants were also invited to complete additional, optional modules at the end of the survey. Given that the sample of parents responding to the sleep module was not the same sample that responded to the physical activity module, for instance, analyses could only be performed on variables within the same module, not across modules. Sixth, the adapted framework for classifying parental support behaviours [34] was developed after the survey questions were designed. Accordingly, only *motivational* and *regulatory* support behaviours were measured and analyzed in the current study. Future studies should consider also including *instrumental* and *conditional* support behaviours to better understand the relationship between parental support and child sleep. Finally, the complex relationship between parental support behaviours, child sleep, and child age is important to consider in future studies. This study does not attempt to form conclusions about the role of child age; for example, we cannot assert that the influence of parental support on child sleep varies across ages. More research on this relationship, with a main focus on child age, is warranted.

## Conclusion

The current study revealed that the contribution of parental support behaviours to predictions of children meeting sleep guidelines varied with the type of support provided, and weekend versus weekday sleep. On weekdays, bedtime rule enforcement—not encouragement—was conducive to children achieving sufficient sleep. Conversely, on weekends, no parental support behaviours predicted children meeting sleep guidelines, highlighting the need for future research on factors influencing child sleep and/or diminishing parental influence on weekends. Given the increasing pervasiveness of electronic media use by children and adolescents, the presence of screens in the bedroom remains a barrier to children achieving sufficient sleep [7, 26, 27]. If parental rules and encouragement to limit bedroom screen use do not support child sleep, efforts should focus on how the effectiveness of these supports can be maximized, and what other elements of the home and bedroom environment should be modified. Moreover, consistent parental support throughout the week and for children of all ages may be an important, yet overlooked, component of overall sleep hygiene. The key recommendation emerging from the present study is for parents to enforce rules about their child’s bedtime on weekdays in order to support them in achieving sufficient sleep. Preventing childhood overweight and obesity necessitates a balance of multiple health behaviours, including physical activity, sedentary behaviour, and sleep. The importance of children getting a good night’s sleep, and the capacity of parents to help them do so, should be emphasized in public health efforts to promote healthy childhoods.

## Abbreviations

CATI: Computer Assisted Telephone Interview
CI: Confidence interval
ROC: Receiver operating characteristic
